# Discordant diagnostic criteria for pneumonia in COPD trials: a review

**DOI:** 10.1183/16000617.0124-2021

**Published:** 2021-11-17

**Authors:** Robert A. Wise, Mona Bafadhel, Courtney Crim, Gerard J. Criner, Nicola C. Day, David M.G. Halpin, MeiLan K. Han, Peter Lange, David A. Lipson, Fernando J. Martinez, Diego J. Maselli, Dawn Midwinter, Dave Singh, Maeva Zysman, Mark T. Dransfield, Richard E.K. Russell

**Affiliations:** 1 Division of Pulmonary and Critical Care Medicine, Johns Hopkins University School of Medicine, Baltimore, MD, USA; 2 Nuffield Dept of Medicine, University of Oxford, Oxford, UK; 3 Clinical Sciences – Respiratory, GSK, Research Triangle Park, NC, USA; 4 Lewis Katz School of Medicine at Temple University, Philadelphia, PA, USA; 5 GlaxoSmithKline, GSK House, Brentford, UK; 6 University of Exeter Medical School, College of Medicine and Health, University of Exeter, Exeter, UK; 7 University of Michigan, Pulmonary & Critical Care, Ann Arbor, MI, USA; 8 Section of Epidemiology, Dept of Public Health, University of Copenhagen, Copenhagen, Denmark; 9 Medical Dept, Herlev and Gentofte Hospital, Herlev, Denmark; 10 Clinical Sciences, GSK, Collegeville, PA, USA; 11 Pulmonary, Allergy and Critical Care Division, Dept of Medicine, Perelman School of Medicine, University of Pennsylvania, Philadelphia, PA, USA; 12 New York-Presbyterian Hospital/Weill Cornell Medical Center, New York, NY, USA; 13 Dept of Medicine, University of Texas Health at San Antonio, San Antonio, TX, USA; 14 Centre for Respiratory Medicine and Allergy, Institute of Inflammation and Repair, Manchester Academic Health Science Centre, The University of Manchester, Manchester University NHS Foundation Hospital Trust, Manchester, UK; 15 Service des Maladies Respiratoires, CHU Bordeaux, Pessac, France; 16 Univ-Bordeaux, Centre de Recherche cardio-thoracique de Bordeaux, U1045, CIC 1401, Pessac, France; 17 Division of Pulmonary, Allergy, and Critical Care Medicine, Lung Health Center, University of Alabama at Birmingham, Birmingham, AL, USA; 18 Affiliation at the time of writing

## Abstract

Inhaled corticosteroids (ICS) have a class effect of increasing pneumonia risk in patients with COPD. However, pneumonia incidence varies widely across clinical trials of ICS use in COPD. This review clarifies methodological differences in defining and recording pneumonia events in these trials and discusses factors that could contribute to the varying pneumonia incidence. Literature searches and screening yielded 40 relevant references for inclusion. Methods used to capture pneumonia events in these studies included investigator-reported pneumonia adverse events, standardised list of signs or symptoms, radiographic confirmation of suspected cases and/or confirmation by an independent clinical end-point committee. In general, more stringent pneumonia diagnosis criteria led to lower reported pneumonia incidence rates. In addition, studies varied in design and population characteristics, including exacerbation history and lung function, factors that probably contribute to the varying pneumonia incidence. As such, cross-trial comparisons are problematic. A minimal set of standardised criteria for diagnosis and reporting of pneumonia should be used in COPD studies, as well as reporting of patients’ pneumonia history at baseline, to allow comparison of pneumonia rates between trials. Currently, within-trial comparison of ICS-containing *versus* non-ICS-containing treatments is the appropriate method to assess the influence of ICS on pneumonia incidence.

## Abstract 2

**Trials of ICS in COPD use varying methods of reporting pneumonia incidence, which influence the interpretation of results. A minimal set of standardised criteria for pneumonia diagnosis would allow comparison of pneumonia incidence between trials.** https://bit.ly/3nbkdoL

## Introduction

Community-acquired pneumonia is one of the most common serious infectious diseases, accounting for almost 1% of all medical admissions [1, 2]. Diagnosis of community-acquired pneumonia using clinical signs and symptoms and laboratory data alone can be inaccurate, due to heterogeneity of clinical presentation, and may be a particular challenge in the presence of chronic respiratory disease [3]. Radiographic confirmation, required to make a definitive diagnosis, is recommended; however, this is often not obtained, particularly in the primary care setting [3]. COPD is a known risk factor for community-acquired pneumonia [4–6]. Furthermore, observational studies suggest that exacerbations caused by pneumonia are associated with an increased risk of intensive care unit admission, need for mechanical ventilation, length of stay and mortality compared with exacerbations not caused by pneumonia in patients with COPD [7, 8].

The Global Initiative for Chronic Obstructive Lung Disease strategy report recommends addition of inhaled corticosteroid (ICS) treatment for patients with COPD with persistent exacerbations despite receiving mono or dual long-acting bronchodilator therapy [9]. These recommendations are made on the basis that addition of ICS reduces exacerbation rates and improves lung function and health status in patients with a history of COPD exacerbations [10–15]. However, ICS have a class effect of increasing the risk of pneumonia in patients with COPD [16–19]. Although this class effect is consistently described in the literature, the rates of pneumonia vary between studies and some analyses have described differing pneumonia rates with different ICS therapies [20–22]. In much of the COPD literature it is unclear how pneumonia events are defined, recorded, graded in terms of severity and adjudicated. In addition, methods for pneumonia capture and assessment can differ between countries; for example, computed tomography is reported to be most frequently used in Japan and the United States [23]. Moreover, factors such as the study design, ascertainment of pneumonia events, patient population and characteristics vary between studies. Heterogeneity in the definition of pneumonia is a potential difficulty for meta-analyses of this outcome.

Given the importance of an accurate estimate of the risk of pneumonia with any given COPD therapy when making treatment decisions, we performed an in-depth review of the literature to examine differences in reporting methodologies. Other factors that may contribute to differing rates of pneumonia reporting in COPD clinical trials of ICS therapy were also considered, such as study design and patient population characteristics.

## Methods

**FIGURE 1:**
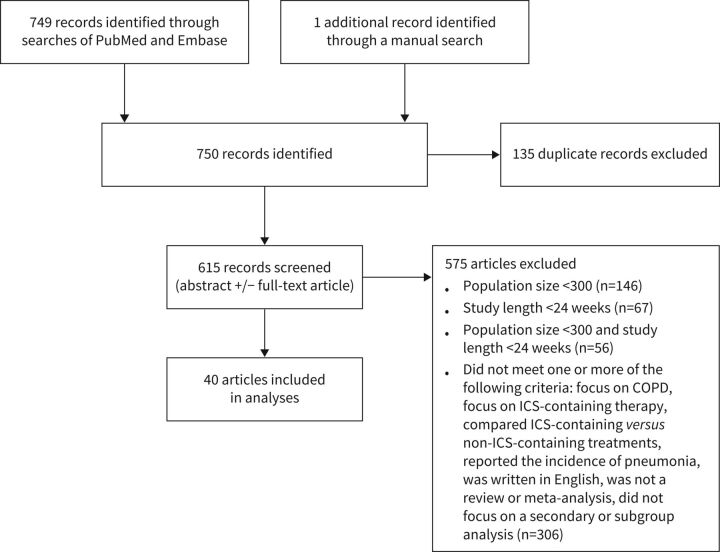
Flowchart describing study selection. ICS: inhaled corticosteroid.

We performed literature searches of the PubMed and Embase databases on 10 August 2020, using the search terms “corticosteroid” OR “glucocorticoid” AND “chronic obstructive lung disease” AND ([controlled clinical trial]/lim OR [randomised controlled trial]/lim) AND [2007–2020]/py) AND (“article”/it OR “article in press”/it). Although the search was done some time prior to publication, updating this search to a more recent date would not, in the opinion of the authors, alter the conclusions. Our initial search yielded 749 publications for review, with 458 from PubMed and 291 from EMBASE. The list of trials retrieved was checked by the authors, and one additional relevant study was added as it was not listed in the initial search results [24]. After eliminating duplicates, 615 results remained. The titles, abstracts and full text of these articles were then screened for relevancy, excluding manuscripts corresponding to studies that were <24 weeks in length, had a population of <300 patients, did not report on patients with COPD or patients receiving ICS-containing therapy, did not compare with a non-ICS-containing treatment, or did not report the incidence of pneumonia (number and/or percentage of patients with pneumonia in each treatment group). Trials included were completed prior to the coronavirus disease 2019 pandemic. Manuscripts focusing on secondary subgroup analyses of clinical trials were also excluded unless they reported the incidence of pneumonia. Following this screening, 40 relevant references remained and were included in the review (figure 1). Each manuscript was examined, and details of the study population and the methodology used to capture pneumonia, as well as the incidence of pneumonia in treatment groups were collected. Risk of pneumonia for each trial was calculated as a fold increase in pneumonia incidence (incidence in treatment arm of interest)/(incidence in comparator arm); however, a limitation of this approach is that not all patients within a study may have been followed for the same length of time. The findings of the analysis are reported in a narrative manner.

## Differences in pneumonia capture and reporting

In clinical studies, a trial investigator would typically report adverse events such as pneumonia as per protocol guidance. These events are then coded to an adverse event term using the preferred terms from the Medical Dictionary for Regulatory Activities (MedDRA) prior to analysing the data. As part of these analyses, pneumonia as an adverse event could be reported as a single preferred term, or as part of a group of several pneumonia-related preferred terms, often labelled adverse events of special interest (AESIs). The grouping of MedDRA preferred terms into AESIs is commonplace in randomised controlled trials (RCTs). However, the reliability and reproducibility of the approach has been questioned, as the definition of which preferred terms comprise a pneumonia AESI could differ between trials. For example, some trials may use Standardised MedDRA Queries (validated, pre-determined sets of MedDRA preferred terms grouped together to enable capture of all plausible events linked to a disease process [25]), and some may define their own set of preferred terms (referred to as sponsor-defined AESI throughout the paper). The latter might include only a few or a large number of preferred terms. Regardless of the number of preferred terms in an AESI, it is important to note that investigator-reported pneumonia-related events might not map to all preferred terms within the AESI. Of note, MedDRA has recently developed a Standardised MedDRA Query for Infective Pneumonia that can be used for analysis of pneumonia-related events [25]. Pneumonias can also be confirmed by chest radiography; these events would not necessarily be dependent on MedDRA terms, as confirmation would be performed based on investigator direction (either investigator decision or driven by protocol); however, they would probably be a subset of a larger set of pneumonia events. Finally, pneumonia events can be subject to adjudication by an independent committee, and instructions as to which events need to be adjudicated can differ between studies (supplementary figure S1).

**FIGURE 2:**
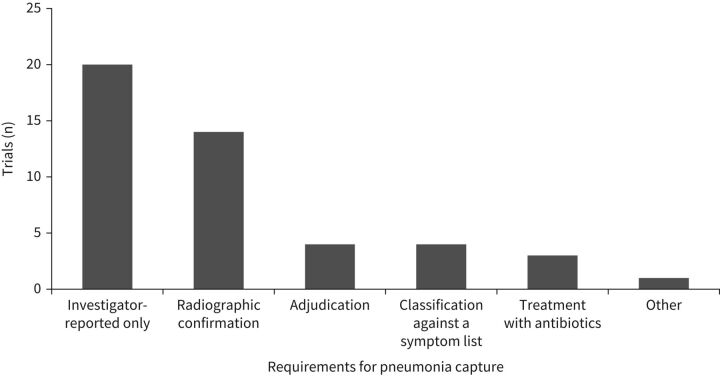
COPD trials by pneumonia capture methodology. Some trials included multiple pneumonia capture methodologies.

There was great variation in the way pneumonia events were captured and recorded in studies of ICS treatment in patients with COPD (table 1). Of the trials identified, 20 evaluated the incidence of investigator-reported pneumonia with no pre-defined, adjudicated or structured approach to diagnosis (figure 2). Radiographic confirmation of suspected pneumonia cases was required for a pneumonia report in 14 trials, a standardised list of clinical signs or symptoms was used in four trials, treatment with antibiotics was required in three trials and confirmation of all pneumonia events by an independent clinical end-point committee was required in four trials (figure 2).

**TABLE 1:** Pneumonia incidence in identified COPD clinical trials, ordered by treatment comparison and pneumonia capture

**Citation**	**Study and study length**	**Study population**	**Pneumonia capture method** **^#^**	**Pneumonia incidence, % (n/N)** **^¶^**		**Increased incidence of pneumonia *versus* comparator arm**	
				**ICS arm**	**Comparator arm**	**Comparison**	**Fold increase in risk** **^+^**
**Studies comparing ICS/LAMA/LABA triple therapy with non-ICS-containing treatment**							
Pneumonia capture: investigator reporting confirmed by radiographic imaging and independent adjudication							
Rabe, 2020 [14]	ETHOS (52 weeks)	Post-bronchodilator FEV_1_ 25–50% pred and ≥1 moderate/severe exacerbation or post-bronchodilator FEV_1_ 50–65% pred and ≥2 moderate or ≥1 severe exacerbation in the year prior to screening CAT total score ≥10	Investigator reported, adjudicated by a clinical end-point independent committee Radiographic imaging compatible with the diagnosis of pneumonia, ≥2 of a list of clinical signs, symptoms or laboratory findings, and treatment with antibiotics and/or antiviral and/or antifungal agents were also required to support the adjudication	BUD/GLY/FOR 320/18/9.6 µg twice daily: 4.2% (90/2144) BUD/GLY/FOR 160/18/9.6 µg twice daily: 3.5% (75/2124) BUD/FOR 320/9.6 µg twice daily: 4.5% (96/2136)	GLY/FOR 18/9.6 µg twice daily: 2.3% (48/2125)	BUD/GLY/FOR 320/18/9.6 µg *versus* GLY/FOR	1.9
						BUD/GLY/FOR 160/18/9.6 µg *versus* GLY/FOR	1.6
						BUD/FOR *versus* GLY/FOR	2.0
Ferguson, 2018 [26]	KRONOS (24 weeks)	Post-bronchodilator FEV_1_ ≥25% and <80% pred CAT total score ≥10	Investigator reported, adjudicated by an independent committee Radiographic imaging compatible with the diagnosis of pneumonia, ≥2 of a list of clinical signs, symptoms or laboratory findings, and treatment with antibiotics and/or antiviral and/or antifungal agents were also required	BUD/GLY/FOR 320/18/9.6 μg twice daily *via* MDI: 1.9% (12/639) BUD/FOR 320/9.6 μg twice daily *via* MDI: 1.9% (6/314) BUD/FOR 400/12 µg twice daily *via* DPI: 1.3% (4/318)	GLY/FOR 18/9.6 μg twice daily *via* MDI: 1.6% (10/625)	BUD/GLY/FOR *versus* GLY/FOR	1.2
						BUD/FOR 320/9.6 µg *via* MDI *versus* GLY/FOR	1.2
						BUD/FOR 400/12 µg *via* DPI *versus* GLY/FOR	0.8
Pneumonia capture: investigator reporting confirmed by radiographic imaging							
Chapman, 2018 [27]	SUNSET (26 weeks)	Post-bronchodilator FEV_1_ ≥40% and <80% pred ≤1 moderate/severe exacerbation in the year prior to screening	Investigator reported Radiographic imaging was required to confirm the diagnosis of pneumonia	TIO 18 µg once daily plus SAL/FP 50/500 µg twice daily: 1.7% (9/526)	IND/GLY 110/50 µg once daily: 1.1% (6/527)	TIO+SAL/FP *versus* IND/GLY	1.5
Lipson, 2018 [13]	IMPACT (52 weeks)	Post-bronchodilator FEV_1_ <50% pred with ≥1 moderate/severe exacerbation in the year prior to screening, or post-bronchodilator FEV_1_ 50–80% pred and ≥2 moderate or ≥1 severe exacerbation in the year prior to screening CAT total score ≥10	Investigator reported Radiographic imaging was required to confirm the diagnosis of pneumonia	FF/UMEC/VI 100/62.5/25 µg once daily: 7.6% (317/4151) FF/VI 100/25 µg once daily: 7.1% (292/4134)	UMEC/VI 62.5/25 µg once daily: 4.7% (97/2070)	FF/UMEC/VI *versus* UMEC/VI	1.6
						FF/VI *versus* UMEC/VI	1.5
Magnussen, 2014 [28]	WISDOM (52 weeks)	Post-bronchodilator FEV_1_ <50% pred ≥1 exacerbation in the year prior to screening	Investigator reported Radiographic imaging was requested when pneumonia was suspected	FP/SAL/TIO (500 µg twice daily/50 µg twice daily/18 µg once daily): 5.8% (72/1243)	SAL/TIO (50 µg twice daily/18 µg once daily): 5.5% (68/1242)	FP/SAL/TIO *versus* SAL/TIO	1.1
Pneumonia capture: investigator reporting							
Papi, 2018 [12]	TRIBUTE (52 weeks)	Post-bronchodilator FEV_1_ <50% pred ≥1 moderate/severe exacerbation in the year prior to screening CAT total score ≥10	Investigator reported	BDP/FOR/GLY 87/5/9 µg twice daily: 3.7% (28/764)	IND/GLY 85/43 µg: 3.6% (27/768)	BDP/FOR/GLY *versus* IND/GLY	1.0
Vestbo, 2017 [29]	TRINITY (52 weeks)	Post-bronchodilator FEV_1_ <50% pred ≥1 moderate/severe exacerbation in the year prior to screening CAT total score ≥10	Investigator reported	BDP/FOR/GLY 100/6/12.5 µg (2 actuations twice daily): 2.6% (28/1077) BDP/FOR 100/6 µg (2 actuations twice daily)+TIO 18 µg (1 actuation once daily): 2.2% (12/537)	TIO 18 µg once daily: 1.8% (19/1076)	BDP/FOR/GLY *versus* TIO	1.5
						BDP/FOR+TIO *versus* TIO	1.3
Jung, 2012 [30]	(24 weeks)	Post-bronchodilator FEV_1_ <65% pred	Investigator reported	TIO 18 µg once daily+FP/SAL 250/50 µg twice daily: 0.9% (2/223)	TIO 18 µg once daily: 0.9% (2/232)	TIO+FP/SAL *versus* TIO	1.0
**Studies comparing ICS/LABA dual therapy with non-ICS-containing treatment**							
Pneumonia capture: investigator reporting and independent adjudication with/without confirmation by radiographic imaging							
Hanania, 2020 [31]	SOPHOS (52 weeks)	Post-bronchodilator FEV_1_ ≥25% and <80% pred ≥1 moderate/severe exacerbation in the year prior to screening CAT total score ≥10	Investigator reported, adjudicated by an independent committee	BUD/FOR 320/10 µg twice daily: 1.6% (10/619) BUD/FOR 160/10 µg twice daily: 2.4% (15/617)	FOR 10 µg twice daily: 2.3% (14/607)	BUD/FOR 320/10 µg *versus* FOR BUD/FOR 160/10 µg *versus* FOR	0.7 1.1
Ferguson, 2018 [32]	TELOS (24 weeks)	Post-bronchodilator FEV_1_ <80% pred CAT total score ≥10	Investigator reported, adjudicated by an independent committee Radiographic imaging compatible with the diagnosis of pneumonia, ≥2 of a list of clinical signs, symptoms or laboratory findings, and treatment with antibiotics and/or antiviral and/or antifungal agents were also required	BUD/FOR 320/10 μg twice daily *via* MDI: 0.8% (5/655) BUD/FOR 160/10 μg twice daily *via* MDI: 1.1% (7/637) BUD 320 µg twice daily *via* MDI: 0.5% (1/206) BUD/FOR 400/12 µg twice daily *via* DPI: 1.4% (3/219)	FOR 10 µg twice daily *via* MDI: 1.4% (9/644)	BUD/FOR 320/10 μg *versus* FOR	0.5
						BUD/FOR 160/10 μg *versus* FOR	0.8
						BUD *versus* FOR	0.3
						BUD/FOR 400/12 µg *via* DPI *versus* FOR	1.0
Pneumonia capture: investigator reporting confirmed by radiographic imaging							
Ferguson, 2017 [33]	RISE (26 weeks)	Post-bronchodilator FEV_1_ ≤70% pred ≥1 moderate/severe exacerbation in the year prior to screening mMRC dyspnoea score ≥2	Investigator reported Radiographic imaging compatible with the diagnosis of pneumonia and ≥2 of a list of clinical signs, symptoms or laboratory findings were also required	BUD/FOR 320/9 µg twice daily: 0.5% (3/605)	FOR DPI 9 µg twice daily: 1.0% (6/613)	BUD/FOR *versus* FOR	0.5
Papi, 2017 [24]	EFFECT (52 weeks)	Post-bronchodilator FEV_1_ ≤50% pred ≥1 moderate/severe exacerbation in the year prior to screening	Investigator reported Radiologically and/or clinically confirmed per British Thoracic Society criteria	FP/FOR 500/20 μg twice daily: 2.9% (17/587) FP/FOR 250/10 μg twice daily: 3.9% (23/588)	FOR 12 µg twice daily: 1.9% (11/590)	FP/FOR 500/20 μg *versus* FOR	1.6
						FP/FOR 250/10 μg *versus* FOR	2.1
Wedzicha, 2016 [34]	FLAME (52 weeks)	Post-bronchodilator FEV_1_ ≥25 to <60% pred ≥1 moderate/severe exacerbation in the year prior to screening mMRC dyspnoea score ≥2	Investigator reported Radiographic imaging was required	FP/SAL 500/50 µg twice daily: 4.8% (80/1680)	IND/GLY 110/50 µg once daily: 3.2% (53/1678)	FP/SAL *versus* IND/GLY	1.5
Ohar, 2014 [35]	NCT01110200 (26 weeks)	Post-bronchodilator FEV_1_ <70% pred Recent exacerbation (≤14 days)	Investigator reported Radiographic imaging was required	FP/SAL 250/50 µg twice daily: 4.1% (13/314)	SAL 50 µg twice daily: 3.1% (10/325)	FP/SAL *versus* SAL	1.3
Vogelmeier, 2013 [36]	ILLUMINATE (26 weeks)	Post-bronchodilator FEV_1_ 40–80% pred 0 moderate/severe exacerbations in the year prior to screening	Investigator reported Radiographic imaging was required	FP/SAL 500/50 µg twice daily: 1.5% (4/264)	IND/GLY 110/50 µg once daily: 0 (0/258)		
Anzueto, 2009 [10]	NCT00115492 (52 weeks)	Post-bronchodilator FEV_1_ ≤50% pred ≥1 moderate/severe exacerbation in the year prior to screening	Investigator reported Radiographic imaging was required	FP/SAL 250/50 µg twice daily: 6.6% (26/394)	SAL 50 µg twice daily: 2.5% (10/403)	FP/SAL *versus* SAL	2.7
Pneumonia capture: investigator reporting							
Suissa, 2018 [37]	Up to 1-year follow-up	Cohort of patients aged ≥55 years with COPD initiating treatment with a LAMA or ICS/LABA during 2002–2015 from the UK's Clinical Practice Research Datalink	Hospital admissions due to severe pneumonia (according to diagnostic codes)	ICS/LABA: 3.1% (380/12 366)	LAMA: 2.3% (279/12 366) of patients	ICS/LABA *versus* LAMA	1.4
Vestbo, 2016 [38], Crim, 2017 [39]	SUMMIT (event driven, common end date of 3 years)	Post-bronchodilator FEV_1_ ≥50% and ≤70% pred History or increased risk of cardiovascular disease mMRC dyspnoea score ≥2	Investigator reported	FF/VI 100/25 µg once daily: 5.7% (237/4140) FF 100 µg once daily: 5.5% (228/4157)	VI 25 µg once daily: 3.9% (163/4140) Placebo: 5.2% (214/4131)	FF/VI *versus* VI	1.5
						FF *versus* placebo	1.1
Vestbo, 2016 [40]	Salford Lung Study (52 weeks)	≥1 COPD exacerbations in the previous 3 years	Investigator reported pneumonia SAEs	FF/VI 100/25 µg once daily: 6.7% (94/1396)	Usual care: 5.9% (83/1403)	FF/VI 100/25 µg *versus* usual care	1.1
Vogelmeier, 2016 [41]	AFFIRM (24 weeks)	Post-bronchodilator FEV_1_ <80% pred CAT total score ≥10	Investigator reported	FP/SAL 500/50 µg twice daily: 1.9% (9/466)	ACL/FOR 400/12 µg twice daily: 0.6% (3/467)	FP/SAL *versus* ACL/FOR	3.2
Zheng, 2015 [42]	NCT01376245 (24 weeks)	Asian patients Post-bronchodilator FEV_1_ ≤70% pred mMRC dyspnoea score ≥2	Investigator reported	FF/VI 50/25 µg: 1.3% (2/160) FF/VI 100/25 µg: 0.6% (1/161) FF/VI 200/25 µg: 3.1% (5/160)	Placebo: 2.5% (4/162)	FF/VI 50/25 µg *versus* placebo	0.5
						FF/VI 100/25 µg *versus* placebo	0.3
						FF/VI 200/25 µg *versus* placebo	1.3
Zhong, 2015 [43]	LANTERN (26 weeks)	Post-bronchodilator FEV_1_ ≥30% and <80% pred ≤1 moderate/severe exacerbation in the year prior to screening mMRC dyspnoea score ≥2	Investigator reported	FP/SAL 500/50 µg: 2.7% (10/369)	IND/GLY 110/50 µg: 0.8% (3/372)	FP/SAL *versus* IND/GLY	3.4
Rossi, 2014 [44]	INSTEAD (26 weeks)	GOLD stage II 0 moderate/severe exacerbation in the year prior to screening	Investigator-reported pneumonia SAEs	FP/SAL 500/50 µg twice daily: 0.7% (2/288)	IND 150 µg once daily: 0 (0/293)	FP/SAL *versus* IND	
Wedzicha, 2014 [45]	FORWARD (48 weeks)	Post-bronchodilator FEV_1_ ≥30% and <50% pred ≥1 moderate/severe exacerbation in the year prior to screening	Investigator reported	BDP/FOR 200/12 µg twice daily: 3.8% (23/601)	FOR 12 µg twice daily: 1.8% (11/596)	BDP/FOR *versus* FOR	2.1
Dransfield, 2013 [46], Crim, 2015 [47]	NCT01009463, NCT01017952 (52 weeks)	Post-bronchodilator FEV_1_ <70% pred ≥1 moderate/severe exacerbation in the year prior to screening	Investigator reported	FF/VI 200/25 µg once daily: 6.8% (55/811) FF/VI 100/25 µg once daily: 6.3% (51/806) FF/VI 50/25 µg once daily: 5.9% (48/820)	VI 25 µg once daily: 3.3% (27/818)	FF/VI 50/25 µg *versus* VI	1.8
						FF/VI 100/25 µg *versus* VI	1.9
						FF/VI 200/25 µg *versus* VI	2.1
Kerwin, 2013 [48]	NCT01053988 (24 weeks)	Post-bronchodilator FEV_1_ ≤70% pred mMRC dyspnoea score ≥2	Investigator reported	FF/VI 100/25 µg once daily: 2.4% (5/206) FF/VI 50/25 µg once daily: 1.5% (3/206) FF 100 µg once daily: 1.9% (4/206)	VI 25 µg once daily: 2.4% (5/205) Placebo: 1.4% (3/207)	FF/VI 100/25 µg *versus* VI	1.0
						FF/VI 50/25 µg *versus* VI	0.6
						FF *versus* placebo	1.3
Martinez, 2013 [49]	NCT01054885 (24 weeks)	Post-bronchodilator FEV_1_ ≤70% pred mMRC dyspnoea score ≥2	Investigator reported	FF/VI 200/25 µg once daily: 2.0% (4/205) FF/VI 100/25 µg once daily: 0.5% (1/204) FF 200 µg once daily: 1.5% (3/203) FF 100 µg once daily: 1.0% (2/204)	VI 25 µg once daily: 1.0% (2/203) Placebo: 0 (0/205)	FF/VI 200/25 µg *versus* VI	2.0
						FF/VI 100/25 µg *versus* VI	0.5
						FF 200 µg *versus* VI	1.5
						FF 100 µg *versus* VI	1.0
Doherty, 2012 [50]	(52 weeks)	Post-bronchodilator FEV_1_ 25–60% pred Symptoms of COPD for ≥24 months prior to enrolment	Investigator reported	MF/FOR 400/10 µg twice daily: 3.1% (7/225) MF/FOR 200/10 µg twice daily: 1.7% (4/239) MF 400 µg twice daily: 2.0% (5/253)	FOR 10 µg twice daily: 1.6% (4/243) Placebo: 0.8% (2/236)	MF/FOR 400/10 µg *versus* FOR	1.9
						MF/FOR 200/10 µg *versus* FOR	1.0
						MF *versus* placebo	2.3
Tashkin, 2012 [51]	NCT00383435 (52 weeks)	Post-bronchodilator FEV_1_ ≥25% and ≤60% pred Symptoms of COPD for ≥24 months	Investigator reported	MF/FOR 400/10 µg twice daily: 1.8% (4/217) MF/FOR 200/10 µg twice daily: 0.5% (1/207) MF 400 µg twice daily: 1.0% (2/210)	FOR 10 µg twice daily: 1.9% (4/209)	MF/FOR 400/10 µg *versus* FOR	1.0
						MF/FOR 200/10 µg *versus* FOR	0.3
						MF *versus* FOR	0.5
Sharafkhaneh, 2012 [52]	NCT00419744 (52 weeks)	Pre-bronchodilator FEV_1_ ≤50% pred ≥1 moderate/severe exacerbation in the year prior to screening	Investigator reported	BUD/FOR 320/9 µg twice daily: 6.4% (26/407) BUD/FOR 160/9 µg: 4.7% (19/408)	FOR 9 µg twice daily: 2.7% (11/403)	BUD/FOR 320/9 µg *versus* FOR	2.3
						BUD/FOR 160/9 µg *versus* FOR	1.7
Calverley, 2010 [53]	NCT00476099 (48 weeks)	Post-bronchodilator FEV_1_ 30–50% pred ≥1 moderate/severe exacerbation in the year prior to screening	Investigator reported	BDP/FOR 200/12 µg twice daily: 2.2% (5/232) BUD/FOR 400/12 µg twice daily: 2.9% (7/238)	FOR 12 µg twice daily: 0.4% (1/233)	BDP/FOR *versus* FOR	5.0
						BUD/FOR *versus* FOR	6.9
Rennard, 2009 [54]	NCT00206167 (52 weeks)	Post-bronchodilator FEV_1_ ≤50% pred ≥1 moderate/severe exacerbation in the year prior to screening mMRC dyspnoea score ≥2	Investigator reported	BUD/FOR 320/9 µg twice daily: 4.0% (20/494) BUD/FOR 160/9 µg twice daily: 3.4% (17/494)	FOR 9 µg twice daily: 3.4% (17/495) Placebo: 5.0% (24/481)	BUD/FOR 320/9 µg *versus* FOR	1.2
						BUD/FOR 160/9 µg *versus* FOR	1.0
Ferguson, 2008 [11]	NCT00144911 (52 weeks)	Post-bronchodilator FEV_1_ ≤50% pred ≥1 moderate/severe exacerbation in the year prior to screening	Investigator reported	FP/SAL 250/50 µg twice daily: 7.4% (29/394)	SAL 50 µg twice daily: 3.9% (15/388)	FP/SAL *versus* SAL	1.9
Tashkin, 2008 [55]	NCT00206154 (26 weeks)	Pre-bronchodilator FEV_1_ ≤50% pred ≥1 moderate/severe exacerbation in the year prior to screening mMRC dyspnoea score ≥2	Investigator reported	BUD/FOR 320/9 µg twice daily: 0.4% (1/277) BUD/FOR 160/9 µg twice daily: 0.7% (2/281) BUD 320 µg twice daily+FOR 9 µg twice daily: 0.7% (2/287) BUD 320 µg twice daily: 1.1% (3/275)	FOR 9 µg twice daily: 0.4% (1/284) Placebo: 0.3% (1/300)	BUD/FOR 320/9 µg *versus* FOR	1.0
						BUD/FOR 160/9 µg *versus* FOR	2.0
						BUD 320 µg+FOR 9 µg *versus* FOR	2.0
						BUD *versus* placebo	3.3
Wedzicha, 2008 [56], Calverley, 2011 [57]	INSPIRE (2 years)	Post-bronchodilator FEV_1_ <50% pred, mMRC dyspnoea score ≥2, clinical history of exacerbations	Investigator reported	FP/SAL 500/50 µg twice daily: 7.6% (50/658)	TIO 18 µg once daily: 3.6% (24/665)	FP/SAL *versus* TIO	2.1
Calverley, 2007 [58], Crim, 2009 [59]	TORCH (3 years) (pneumonia incidences from a *post hoc* analysis focusing on pneumonia are reported here)	Pre-bronchodilator FEV_1_ <60% pred	Investigator reported	FP 500 µg twice daily: 14.4% (224/1552) FP/SAL 500/50 µg twice daily: 16.0% (248/1546)	SAL 50 µg twice daily: 10.5% (162/1542) Placebo: 9.0% (139/1544)	FP/SAL *versus* SAL	1.5
						FP *versus* placebo	1.6

ICS: inhaled corticosteroid; LAMA: long-acting muscarinic antagonist; LABA: long-acting β_2_-agonist; FEV_1_: forced expiratory volume in 1 s; CAT: COPD Assessment Test; BUD: budesonide; GLY: glycopyrronium; FOR: formoterol; MDI: metered-dose inhaler; DPI: dry powder inhaler; TIO: tiotropium; SAL: salmeterol; FP: fluticasone propionate; FF: fluticasone furoate; UMEC: umeclidinium; VI: vilanterol; BDP: beclomethasone dipropionate; mMRC: modified Medical Research Council; SAE: serious adverse event; ACL: aclidinium; IND: indacaterol; GOLD: Global Initiative for Chronic Obstructive Lung Disease; MF: mometasone furoate. ^#^: when a study uses different sets of criteria to define pneumonia and reports different pneumonia incidences when using these different criteria, only the pneumonia incidence for the most stringent pneumonia capture method is reported. The corresponding method is summarised in the “pneumonia capture method” column; ^¶^: N is the total number of patients in the subgroup of interest, n the number of patients with events; ^+^: fold increase calculated as (incidence in treatment arm of interest)/(incidence in comparator arm).

### Investigator-reported pneumonia

In total, 22 (55.0%) studies relied on investigator assessment of adverse events to report pneumonia without requiring radiographic confirmation, the presence of standardised clinical signs or symptoms, treatment with antibiotics or confirmation by an independent clinical end-point committee (table 1).

A pre-specified definition of pneumonia was not included in the protocol of the 3-year TORCH study, as the increased risk of pneumonia in patients treated with fluticasone propionate (FP)/salmeterol (SAL) or FP *versus* SAL or placebo was unexpected at that time [58]. Investigator-reported on-treatment pneumonia was grouped as a sponsor-defined AESI comprising 15 pneumonia-related MedDRA preferred terms [58, 59]. Investigators were not required to provide supporting evidence, such as a chest radiograph or further laboratory tests with respect to pneumonia events [58, 59]. In a *post hoc* analysis of TORCH, these on-treatment pneumonia AESIs over the 3-year study period were reported for 16.0% (n=248), 14.4% (n=224) and 10.5% (n=162) of patients treated with FP/SAL, FP and SAL, respectively, representing a 1.5-fold increased risk of pneumonia with FP/SAL *versus* SAL (table 1) [58, 59].

In the phase III 52-week IMPACT study, patients were randomised 2:2:1 to treatment with fluticasone furoate (FF), umeclidinium (UMEC) and vilanterol (VI), FF/VI or UMEC/VI, administered in a single inhaler [13]. Investigator-reported pneumonia was evaluated as a sponsor-defined AESI comprising 70 pneumonia-related MedDRA preferred terms. Events reported as pneumonia by the investigator required confirmation by the presence of new infiltrate(s) on a chest radiograph and at least two clinical signs from a pre-defined list. Pneumonia events were reported for 7.6% (n=317), 7.1% (n=292) and 4.7% (n=97) of patients treated with FF/UMEC/VI, FF/VI and UMEC/VI, respectively [13].

Dransfield *et al*. [46] reported the findings of two replicate 1-year trials that compared treatment with VI 25 µg combined with FF 50, 100 or 200 µg *versus* treatment with VI 25 µg alone. Pneumonia was assessed as a sponsor-defined AESI of 48 pneumonia-related MedDRA preferred terms [47], and was reported for 5.9% (n=48), 6.3% (n=51), 6.8% (n=55) and 3.3% (n=27) of patients assigned to these treatment groups, respectively, representing a 1.8–2.1-fold increased risk of pneumonia with FF/VI *versus* VI [46]. In contrast, in the 1-year TRIBUTE study, comparing beclomethasone dipropionate (BDP)/glycopyrrolate (GLY)/formoterol (FOR) with indacaterol (IND)/GLY, pneumonia was assessed using a narrower sponsor-defined AESI of seven pneumonia-related MedDRA preferred terms. Pneumonia was reported in 3.7% (n=28) of patients treated with BDP/GLY/FOR and 3.6% (n=27) of those treated with IND/GLY; no increased risk of pneumonia was seen with this triple therapy *versus* the long-acting β_2_-agonist (LABA)/long-acting muscarinic antagonist (LAMA) combination [12]. In contrast, Sharafkhaneh *et al*. [52] reported pneumonia, assessed using six preferred terms, for 6.4% (n=26) and 4.7% (n=19) of patients treated with BDP/FOR 320/9 µg and BDP/FOR 160/9 µg, demonstrating a 2.3- and 1.7-fold increased risk of pneumonia, respectively, *versus* FOR 9 µg (2.7%, n=11).

It should be noted that in studies that only used investigator reporting of adverse events and AESIs to capture pneumonia, differences in the incidence of pneumonia events were also influenced by other factors, such as patient population and length of study, in addition to the number of preferred terms used. Indeed, increasing the number of preferred terms used to define pneumonia is likely to capture less frequently reported terms and therefore may not have as big an impact on the incidence reported as other factors, such as patient population and length of study. Nonetheless, while these studies varied in design and duration, they tended to report marginally higher incidences of pneumonia than those in which additional methods were used to guide reporting of pneumonias, such as the presence of standardised symptoms, radiological confirmation or adjudication by an independent clinical end-point committee.

### Radiographic imaging

In addition to investigator reporting of pneumonia adverse events, some RCTs have required supporting radiographic imaging for a report of pneumonia to be made; in this review 33.3% (12 out of 36) trials required supporting radiographic confirmation. It is important to note that the accuracy of supportive radiographic imaging can differ depending on where and how it is conducted, the clinical scenario of each individual patient and whether the radiographic assessment is done by a single radiologist or a panel who review the images in a blinded fashion. Additionally, it is often not clear whether the report of pneumonia in a given study followed radiological reporting alone, or included physician review. In addition, radiographic imaging practices vary by country, with computed tomography most frequently used in Japan and the United States [23, 60]. Furthermore, some trial protocols, such as the one for IMPACT, asked for chest radiography to be conducted within 48 h of both pneumonia and exacerbation events [13, 61], which may increase the identification of pneumonia that otherwise would go unrecognised. Conversely, this additional diagnostic criterion of chest radiography may contribute to a lower reported incidence of pneumonia compared with relying on investigator reports of pneumonia adverse events alone. For example, while the 1-year EFFECT study reported radiologically and/or clinically defined pneumonia per British Thoracic Society criteria for 1.9%, 3.9% and 2.9% of patients treated with FOR 12 µg, FP/FOR 250/10 µg or FP/FOR 500/20 µg, respectively, radiological confirmation alone led to a slight decrease in reported pneumonia incidence in all treatment groups to 1.5%, 3.2% and 2.4%, respectively [24]. Similarly, in two 1-year trials that compared the efficacy and safety of FF/VI *versus* VI, incidences of pneumonia events with compatible parenchymal infiltrates shown by chest radiograph, over-read by a central laboratory, were lower in all treatment groups (FF/VI 50/25 µg 3.9%; FF/VI 100/50 µg 4.0%; FF/VI 200/25 µg 4.6%; and VI 25 µg 1.8%) compared with reported pneumonia defined as an AESI (5.9%, 6.3%, 6.8% and 3.3%, respectively) [46, 47]. The lower reported incidence of radiologically confirmed pneumonia in these studies, compared with investigator reporting alone, demonstrates the importance of accurate and consistent pneumonia reporting within these trials to allow evaluation of the incidence of pneumonia across different treatments. The reasons behind these observations are complex and multifactorial and could represent true misclassification, or could be due to factors such as availability of imaging and interpretation.

### Standardised symptoms and/or independent adjudication

A further approach to define pneumonia is the use of a standardised list of clinical signs and symptoms, and this method has been used in several studies included in our review. Some of these studies also required treatment with antibiotics and/or antiviral and/or antifungal agents to define pneumonia, and/or adjudication by an independent clinical end-point committee. Such adjudication committees often have access to a patient's full medical record and may, in some cases, adjudicate an event as pneumonia even if the initial chest radiograph was clear.

In the RISE study, confirmed pneumonias were defined by the presence of a new infiltrate on a chest radiography as well as evidence of two or more of a standardised list of clinical signs and symptoms [33]. The reported incidence of pneumonia was 0.5% (n=3) of patients treated with budesonide (BUD)/FOR and 1.0% (n=6) of patients treated with FOR alone [33]. In the SOPHOS study, all potential pneumonia cases were adjudicated by an external clinical end-point committee [31]. Pneumonia was reported for 1.6% (n=10) and 2.4% (n=15) of patients treated with BUD/FOR 320/10 µg and 160/10 µg, respectively, compared with 2.3% (n=14) of patients treated with FOR 10 µg [31]. The phase III ETHOS and KRONOS studies, which investigated single-inhaler triple therapy with BUD/GLY/FOR *versus* BUD/FOR and GLY/FOR, required clinical diagnosis by the investigator, compatible chest imaging, two or more of a standardised list of clinical signs, symptoms or laboratory findings and treatment with antibiotics and/or antiviral and/or antifungal agents as part of the definition of pneumonia [14, 26]. All pneumonia adverse events in the ETHOS and KRONOS studies were adjudicated by an independent clinical end-point committee. Across these studies, confirmation of initial reported pneumonias by the independent clinical end-point committee led to a reduction in the reported rates (table 2) [14, 26]. In the TELOS study, pneumonia was defined according to clinical diagnosis by the investigator alongside compatible chest imaging, treatment with antibiotics and/or appropriate antiviral or antifungal agents, and two or more of a list of respiratory symptoms [32]. Similar to ETHOS and KRONOS, an independent clinical end-point committee reviewed all adverse events reported as pneumonia. The incidence of pneumonia was low, with adjudicated pneumonia reported for 0.8–1.4% of patients treated with BUD/FOR. Overall, these studies further demonstrate the importance of taking into account the way that pneumonia events are reported when reviewing pneumonia data from COPD clinical trials.

**TABLE 2:** Pneumonia incidence determined by clinical end-point committee

**Study and treatment group**	**Patients, n**	**Pneumonia events submitted to clinical end-point committee, n (%)**	**Pneumonia events confirmed by clinical end-point committee, n (%)**
**ETHOS [14]**			
BUD/GLY/FOR (160/18/9.6 µg)	2124	100 (4.7)	75 (3.5)
BUD/GLY/FOR (320/18/9.6 µg)	2144	115 (5.4)	90 (4.2)
BUD/FOR (320/9.6 µg)	2136	118 (5.5)	96 (4.5)
GLY/FOR (18/9.6 µg)	2125	66 (3.1)	48 (2.3)
**KRONOS [26]**			
BUD/GLY/FOR (320/18/9.6 µg; *via* MDI)	639	16 (2.5)	12 (1.9)
BUD/FOR (320/9.6 µg; *via* MDI)	314	7 (2.2)	6 (1.9)
BUD/FOR (400/12 µg; *via* DPI)	318	6 (1.9)	4 (1.3)
GLY/FOR (18/9.6 µg; *via* MDI)	625	11 (1.8)	10 (1.6)

BUD: budesonide; GLY: glycopyrronium; FOR: formoterol; MDI: metered dose inhaler; DPI: dry powder inhaler.

### Real-world evidence

Our literature search also identified two real-world evidence studies reporting the incidence of pneumonia in patients treated with ICS. Notably, both reported the incidence of serious pneumonia events rather than all pneumonia, as the study protocol restricted the collection of safety data to serious events only, with Suissa *et al*. [37] defining serious pneumonia as incidences of pneumonia requiring hospitalisation, and Vestbo *et al*. [40, 62] defining it as the proportion of participants that experienced pneumonia that resulted in death, was life threatening, required hospitalisation or prolongation of hospitalisation. These events have also been considered a significant medical event in the investigator's judgement. Suissa *et al*. [37] performed a retrospective, observational cohort study to evaluate the effectiveness of ICS/LABA *versus* LAMA in patients with COPD using the UK Clinical Practice Research Datalink and propensity score matching. The study reported annual rates of hospital admission due to serious pneumonia of 7.6 and 5.2 per 100 person-years, respectively [37]. The Salford Lung Study evaluated the effectiveness of FF/VI in clinical practice, and reported pneumonia serious adverse events (SAEs) as part of its safety assessments by means of monitoring of electronic health records [40]. The study reported pneumonia SAEs in 6.7% (n=94) of patients on FF/VI *versus* 5.9% (n=83) of patients who continued usual care across a 1-year period [40]. However, information on how these cases were diagnosed is not provided in the publication. Additionally, caution should be taken in the interpretation of these results, as pneumonia events were reported based on the randomised treatment arm and while patients randomised to FF/VI were allowed to switch to usual care, switching from usual care to FF/VI was not permitted [40].

It should also be noted that the accuracy of pneumonia as a clinically coded diagnosis in usual clinical practice is low. Studies performed in UK hospitals have shown that up to 50% of all diagnoses in discharge summaries are inaccurate and >30% of pneumonia clinical coding is unreliable [63–65]. In these studies, 27–47% of coded pneumonia diagnoses did not have any evidence of consolidation in chest radiographs [63, 64], suggesting that clinical coding alone may be overestimating the incidence of pneumonia. This supports the lower incidence of pneumonia seen in studies that used chest radiography or adjudication to confirm pneumonia events, and also highlights a need to improve pneumonia diagnoses within usual clinical practice in addition to clinical studies.

## Distinguishing between pneumonia and COPD exacerbations

A key clinical challenge regarding pneumonia capture in patients with COPD is distinguishing between pneumonia and COPD exacerbations due to the overlap in clinical presentation [8]. COPD trials evaluating ICS-containing therapy are likely to have a patient population that is predisposed to exacerbations and are often enriched for this trait, as the addition of ICS treatment is recommended for patients with COPD with persistent exacerbations despite long-acting bronchodilator therapy [9]. During an exacerbation, chest radiographs may demonstrate pulmonary infiltrates [7], potentially resulting in an increase in the reported rate of pneumonia in the absence of elevated white blood cell count or fever. In two replicate 1-year trials comparing treatment with FF/VI *versus* VI alone, pneumonia was reported as an adverse event for only 72 (60%) of the 120 exacerbation events for which an infiltrate was detected *via* chest radiography, demonstrating that pneumonia may go unreported in the context of a COPD exacerbation event [47]. This clinical overlap of symptoms observed between COPD exacerbations and pneumonia, as well as the potential for patients to experience an exacerbation in the presence of comorbid pneumonia, poses a further challenge for the clinical diagnosis of pneumonia [66]. In addition, treatment decisions are not a reliable criterion for distinguishing between a pneumonia event and an exacerbation, as both are treated with antibiotics [67]. Furthermore, these patients are also often treated with systemic corticosteroids, which are known to elevate white blood cells and suppress fever upon initiation and could further confuse the distinction between pneumonia and an exacerbation [68]. There are regional cultural differences in acceptability of the term exacerbation *versus* pneumonia, and diagnosis may influence access to antibiotics [69–71]. Differentiation is also confounded by an association between unresolved exacerbations and pneumonia, which has been reported for FP treatment [57]. Finally, patients with COPD, particularly those with severe disease, may be at risk of cardiac and other pulmonary conditions that may be misclassified as exacerbations or pneumonia [72]. As such, the accurate capture of pneumonia is further complicated by the clinical characteristics of COPD.

## Differences in study design and population characteristics

While the method used for diagnosing pneumonia can affect the reported incidence in COPD trials, this is not the only aspect of a study that can affect pneumonia incidence rates. Particulars of the study design, such as the length of the study, as well as the characteristics and demographics of the patients enrolled can play an important part in the reported incidence of pneumonia.

### Study design

Study design is likely to contribute to the varied reported incidences of pneumonia across the included studies. For example, the TORCH study had a 3-year treatment period and the INSPIRE study had a 2-year treatment period [56, 58], whereas SUMMIT was event driven with a median treatment duration of 1.8 years [38]. Other studies such as KRONOS and SUNSET were 24 or 26 weeks long (table 1) [26, 27]. With an event occurring as infrequently as pneumonia, these differences in study length impact the proportion of patients who experience the event during the course of the study. In addition, pneumonia has been shown to follow seasonal patterns, with relatively high pneumonia rates reported in winter; therefore, seasonality probably contributes to differences in pneumonia incidence according to study timing and length [73]. Furthermore, elements such as the design of the period prior to initiating study treatment are likely to affect the risk of patients experiencing pneumonia. For example, patients in IMPACT continued on their own medication for 2 weeks prior to initiating study treatment [13], whereas patients in the FLAME trial had a 4-week run-in period during which their own medication, including ICS, was stopped, and they received daily tiotropium (TIO) treatment, potentially removing patients from the trial who were most likely to benefit from ICS [34]. These elements of the study design are important considerations when interpreting the reported pneumonia incidence.

### Population characteristics

A number of risk factors for pneumonia have been identified for patients with COPD, including older age, prior COPD exacerbation or respiratory tract infection, low body mass index, dyspnoea, presence of bronchiectasis or history of asthma, history of pneumonia, low blood eosinophil count, active smoking and severe airflow limitation [6, 20, 59, 73, 74]. A recent meta-analysis reported a significant difference in the risk of pneumonia according to the severity of COPD [21]. An increased incidence of investigator-reported pneumonia in patients in Asia compared with those in non-Asia regions was reported in the IMPACT study [75]. Pneumonia may be more common in Asian patients; however, differences in diagnostic processes, with more events diagnosed with chest radiography or computed tomography in Asia, may also contribute to the differences in pneumonia rates [75]. Inclusion of patients with these characteristics in study populations is highly likely to contribute to the variation in the reported rates of pneumonia between studies.

### Exacerbation history

The phase III KRONOS and ETHOS studies both investigated single-inhaler triple therapy with BUD/GLY/FOR *versus* BUD/FOR and GLY/FOR and used the same set of criteria for pneumonia capture and assessment. However, the two studies had different inclusion criteria, and therefore different patient populations. Patients enrolled in the 24-week KRONOS study were not required to have experienced a COPD exacerbation and overall 74% (n=1411) of patients experienced no moderate/severe exacerbations in the year prior to screening [26]. The risk of adjudicated pneumonia events with BUD/GLY/FOR 320/18/9.6 µg, BUD/FOR 320/9.6 µg and BUD/FOR 400/12 µg, increased by 1.2-, 1.2- and 0.8-fold, respectively, *versus* GLY/FOR 18/9.6 µg treatment (table 1) [26]. In contrast, in the 52-week ETHOS study patients were required to have experienced at least one moderate/severe COPD exacerbation (if forced expiratory volume in 1 s (FEV_1_) <50% predicted) or at least two moderate or at least one severe exacerbation (if FEV_1_ ≥50% pred) in the year prior to screening [14]. In total, 57% (n=4810) of patients experienced two or more moderate/severe exacerbations and 21% (n=1801) of patients experienced one or more severe exacerbations in the year before screening [14]. Risk of adjudicated pneumonia events increased by 1.9-, 1.6- and 2.0-fold in patients treated with BUD/GLY/FOR 320/18/9.6 µg, BUD/GLY/FOR 160/18/9.6 µg and BUD/FOR 320/9.6 µg, respectively, *versus* GLY/FOR 18/9.6 µg (table 1) [14]. This increased risk of pneumonia with ICS- *versus* non-ICS-containing treatment *versus* those reported for the low-exacerbating KRONOS population support the contribution of a prior history of exacerbation to varying pneumonia incidence across studies.

Similarly, other studies conducted in high-exacerbating populations have reported higher incidences of pneumonia than those including low-exacerbating populations. The LANTERN study enrolled patients with a history of one or fewer moderate/severe exacerbation in the previous year [43]. The incidence of investigator-reported pneumonia was 3.4-fold higher in patients treated with FP/SAL (2.7%, n=10) *versus* IND/GLY (0.8%, n=3) [43]. The ILLUMINATE and INSTEAD studies required patients to have experienced no moderate/severe exacerbations in the year prior to screening [36, 44]. These studies reported very low incidences of pneumonia: 1.5% (n=4) of patients treated with FP/SAL and no patients treated with IND/GLY in ILLUMINATE reported radiographically confirmed pneumonia; 0.7% (n=2) of patients treated with FP/SAL and no patients treated with IND in INSTEAD reported pneumonia SAEs [36, 44]. In contrast, Ohar *et al*. [35] conducted a randomised, parallel-group study comparing FP/SAL with SAL monotherapy for the treatment of patients with COPD who had experienced an exacerbation within the previous 14 days. Treatment with FP/SAL was associated with a 1.3-fold increased risk of radiographically confirmed pneumonia *versus* SAL, with pneumonia reported for 4.1% (n=13) and 3.1% (n=10) of patients, respectively [35].

### Lung function

The identified studies recruited patients with a wide range of airflow limitation severity. Overall, the literature search identified 12 studies that recruited only patients with severe airflow limitation (post-bronchodilator FEV_1_ ≤50% pred) [10–12, 24, 28, 29, 45, 52–56]. The incidence of pneumonia for patients treated with ICS-containing therapies in these studies ranged from 0.4% to 7.6%, while the incidence for patients treated with non-ICS-containing therapies in these studies ranged from 0.3% to 5.5% [10–12, 24, 28, 29, 45, 52–56].

The SUNSET study recruited patients with moderate to severe airflow limitation (post-bronchodilator FEV_1_ ≥40% pred and <80% pred), with a mean FEV_1_ of 57% pred at baseline [27]. Pneumonia was reported for a relatively small proportion of patients: 1.7% (n=9) of patients treated with triple therapy with FP/SAL and TIO and 1.1% (n=6) of patients treated with IND/GLY [27]. This represents a 1.5-fold increased risk of pneumonia with ICS/LABA/LAMA *versus* LABA/LAMA treatment in this study. The ILLUMINATE trial also included patients with a post-bronchodilator FEV_1_ of 40–80% pred [36]. The study population had a mean FEV_1_ of 51% pred at baseline, and a relatively small proportion of patients experienced radiologically confirmed pneumonia (FP/SAL: 1.5%, n=4; IND/GLY: 0) [36]. By comparison, in patients from the ETHOS study, who had a post-bronchodilator FEV_1_ of 43.1–43.6% pred, confirmed pneumonia was reported in 3.5–4.5% of patients receiving ICS during the study *versus* 2.3% for patients not receiving ICS [14]. These relatively high proportions of patients with confirmed pneumonia in the ETHOS study may reflect the relatively high proportion of patients who had severe airflow limitation, as ∼70% of patients had a post-bronchodilator FEV_1_ <50% pred [14]. The SUMMIT study recruited only patients with moderate airflow limitation (FEV_1_ ≥50% pred and ≤70% pred), yet reported pneumonia in a relatively high proportion of patients: 5.7% (n=237), 5.5% (n=228), 3.9% (n=163) and 5.2% (n=214) of patients treated with FF/VI, FF/VI, VI and placebo, respectively. However, it is worth noting that SUMMIT recruited patients with a history, or at increased risk, of cardiovascular disease and the mean study exposure was 1.8 years, which may account for the difference observed between the SUMMIT and ETHOS trials. These studies highlight the complexities of measuring the incidence of pneumonia and demonstrate the need to consider all study characteristics when interpreting the findings with regard to pneumonia incidence in different trials.

## Towards a standardised definition of pneumonia for COPD clinical trials

This review has shown that pneumonia in COPD trials has been captured using a variety of methods, including investigator reporting of pneumonia adverse events or confirmation with radiographic imaging with or without the requirement for specific clinical symptoms or laboratory findings, antibiotic and/or antiviral and/or antifungal treatment, or adjudication by an independent committee. Therefore, meaningful comparisons of pneumonia rates between individual trials cannot be made. Furthermore, these results can only be interpreted with knowledge of the study design, patient population, the countries where the study was based and how the diagnosis of pneumonia was made. This adds a burden to readers who may choose to accept a figure given in an abstract rather than look further.

We propose that a minimal set of standardised criteria for the diagnosis of pneumonia should be used in COPD clinical trials that include pneumonia as an outcome or as an expected adverse event (supplementary table S1). This would allow comparison of pneumonia rates between COPD trials, which is of critical importance as pneumonia is a known safety signal for ICS-containing treatments. As mentioned previously, MedDRA has recently developed a Standardised MedDRA Query for Infective Pneumonia that could be used to standardise the capture and analysis of pneumonia-related events in clinical trials [25]. As history, physical examination and laboratory data are inadequate to either exclude or diagnose community-acquired pneumonia [76], suspected pneumonia should be confirmed using a chest radiograph or computed tomography scan. Diagnostic criteria as outlined by the British Thoracic Society, which are straightforward and emanate from an authoritative professional society, would make a good base for standardisation [77], while a requirement for all pneumonia cases to be adjudicated by an independent committee with explicitly agreed-upon criteria would also improve the ability to compare pneumonia incidences between studies. Furthermore, the history of pneumonia within the patient population at baseline should be reported for all trials as well as the criteria for the diagnosis of pneumonia. In addition, the use of individual participant data from randomised controlled COPD trials could be used to derive standardised outcome definitions across trials [78]. It should be reiterated that this set of recommendations refers to the diagnosis of pneumonia in COPD clinical trials and not the diagnosis of pneumonia in usual clinical practice. However, our recommendations could be extended beyond pneumonia in patients with COPD and applied to any clinical trial in which pneumonia is a reported outcome.

## Conclusions

Pneumonia is a known class effect of ICS in patients with COPD [19]. Clinical trials that have examined the long-term use of ICS in patients with COPD have reported large differences in the rates of pneumonia. Differences in the prevalence of risk factors for pneumonia between study populations may contribute to this variation, and to the ability to detect differences in pneumonia rates and their magnitude. Furthermore, analysis of pneumonia as a group of pneumonia-related terms and less stringent confirmation criteria may lead to relatively greater reported rates of pneumonia than the use of a narrower set of terms and adjudication by an independent clinical end-point committee. Across-study comparisons of pneumonia incidence are problematic and the potential confounding factors, such as population risk of pneumonia and variation in pneumonia definitions, should be carefully considered. Therefore, across-study comparisons of pneumonia incidence should be avoided. Greater transparency in the reporting of the methods used to define pneumonia is critical to allow pneumonia rates to be evaluated in the context of other studies. As such, within-trial comparisons of ICS-containing *versus* non-ICS-containing treatments are the only appropriate method to assess the influence of ICS on pneumonia incidence. Importantly, evaluation of the overall risk–benefit profile of the use of ICS in patients with COPD should include the impact on exacerbation risk, lung function, health-related quality of life and mortality as well as the incidence of pneumonia.

A minimal, standardised set of criteria for the diagnosis of pneumonia should be used in studies with pneumonia as an outcome or as an expected adverse event. The criteria used should be clearly defined in the study protocol and the resulting publication.

## Supplementary material

10.1183/16000617.0124-2021.Supp1 **Please note:** supplementary material is not edited by the Editorial Office, and is uploaded as it has been supplied by the author.
Supplementary material ERR-0124-2021.SUPPLEMENT
