# Supplementary material for: Discordant diagnostic criteria for pneumonia in COPD trials: a review
Source: Eur Respir Rev. 2021 Nov 17;30(162):210124. doi: 10.1183/16000617.0124-2021 (PMC9488621; doi:10.1183/16000617.0124-2021)
Supplement: Supplementary file 1 [file ERR-0124-2021.SUPPLEMENT.pdf]

***Supplementary Table 1. Proposed key set of standardised criteria for pneumonia diagnosis***

| |
|---|
| • Diagnostic criteria as outlined by the British Thoracic Society diagnosis criteria should be used as a basis for standardisation [1]<br>• Suspected pneumonia should be confirmed using a chest x-ray or computed tomography scan<br>• If pneumonia is an adverse event of special interest, all pneumonia cases should be adjudicated by an independent committee with clearly defined criteria<br>• The history of pneumonia within the patient population at baseline should be reported for all trials |

***Supplementary Figure 1. Reporting methods and analysis of pneumonia adverse events***

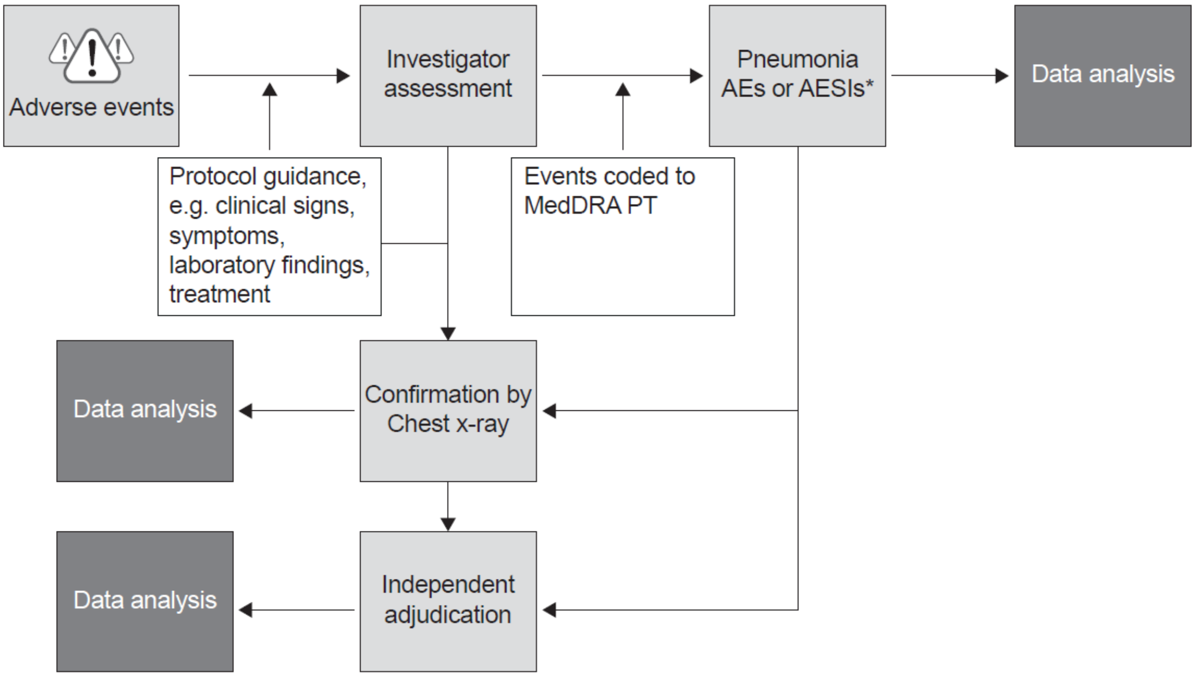


*AESIs are groups of related AEs.

AE, adverse event; AESI, adverse event of special interest; MedDRA, Medical Dictionary for Regulatory Activities; PT, Preferred Term.

**References**


1. Lim WS, Baudouin SV, George RC, Hill AT, Jamieson C, Le Jeune I, Macfarlane JT, Read RC, Roberts HJ, Levy ML, Wani M, Woodhead MA. BTS guidelines for the management of community acquired pneumonia in adults: update 2009. Thorax 2009; 64 Suppl 3: iii1-55.
